# Supplementary material for: Ghost admixture in eastern gorillas
Source: Nat Ecol Evol. 2023 Jul 27;7(9):1503–14. doi: 10.1038/s41559-023-02145-2 (PMC10482688; doi:10.1038/s41559-023-02145-2)
Supplement: Supplementary file 2 — Reporting Summary [file 41559_2023_2145_MOESM2_ESM.pdf]

nature portfolio

Corresponding author(s): Martin Kuhlwilm

Last updated by author(s): Jun 1, 2023

Reporting Summary

Nature Portfolio wishes to improve the reproducibility of the work that we publish. This form provides structure for consistency and transparency in reporting. For further information on Nature Portfolio policies, see our Editorial Policies and the Editorial Policy Checklist.

Statistics

For all statistical analyses, confirm that the following items are present in the figure legend, table legend, main text, or Methods section.

n/a	Confirmed	
☐	☒	The exact sample size (*n*) for each experimental group/condition, given as a discrete number and unit of measurement
☒	☐	A statement on whether measurements were taken from distinct samples or whether the same sample was measured repeatedly
☒	☐	The statistical test(s) used AND whether they are one- or two-sided<br>*Only common tests should be described solely by name; describe more complex techniques in the Methods section.*
☒	☐	A description of all covariates tested
☐	☒	A description of any assumptions or corrections, such as tests of normality and adjustment for multiple comparisons
☐	☒	A full description of the statistical parameters including central tendency (e.g. means) or other basic estimates (e.g. regression coefficient) AND variation (e.g. standard deviation) or associated estimates of uncertainty (e.g. confidence intervals)
☒	☐	For null hypothesis testing, the test statistic (e.g. *F*, *t*, *r*) with confidence intervals, effect sizes, degrees of freedom and *P* value noted<br>*Give P values as exact values whenever suitable.*
☐	☒	For Bayesian analysis, information on the choice of priors and Markov chain Monte Carlo settings
☒	☐	For hierarchical and complex designs, identification of the appropriate level for tests and full reporting of outcomes
☒	☐	Estimates of effect sizes (e.g. Cohen's *d*, Pearson's *r*), indicating how they were calculated

Our web collection on statistics for biologists contains articles on many of the points above.

Software and code

Policy information about availability of computer code

Data collection	no software was used
Data analysis	bcftools 1.6/1.12/1.14, bedtools 2.26.0, bedops, gcc 6.3.0, hmmix, jvarkit, ms, msprime, openssl 1.0.2q, perl, python 2.7.11/2.7.14/2.7.17, R 3.2.0/3.4.0/4.0.1, samtools 1.0, sstar, tabix 0.2.6, Variant Effect Predictor 83, vcftools 0.1.15, VolcanoFinder 1.0, xz 5.2.2, zlib 1.2.8, WebGestaltR

For manuscripts utilizing custom algorithms or software that are central to the research but not yet described in published literature, software must be made available to editors and reviewers. We strongly encourage code deposition in a community repository (e.g. GitHub). See the Nature Portfolio guidelines for submitting code & software for further information.

Data

Policy information about availability of data

All manuscripts must include a data availability statement. This statement should provide the following information, where applicable:

- Accession codes, unique identifiers, or web links for publicly available datasets
- A description of any restrictions on data availability
- For clinical datasets or third party data, please ensure that the statement adheres to our policy

The six newly sequenced eastern gorilla samples are publicly available in the European Nucleotide Archive (ENA) under the project number: PRJEB12821. ENA accession numbers for all samples used in this study are given in Table S1. The human reference genome (hg19) and the rhesus macaque reference genome

(Mmul10/rheMac10) were downloaded from https://hgdownload.soe.ucsc.edu/goldenPath/. Pre-calculated GERP scores for hg19 were accessed from (http://mendel.stanford.edu/SidowLab/downloads/gerp/) and LINSIGHT scores for hg19 from (https://rdrr.io/github/rcastelo/GenomicScores/src/inst/scripts/make-data_linsight.UCSC.hg19.R).

Human research participants

Policy information about studies involving human research participants and Sex and Gender in Research.

Reporting on sex and gender	Not applicable
Population characteristics	Not applicable
Recruitment	Not applicable
Ethics oversight	Not applicable

Note that full information on the approval of the study protocol must also be provided in the manuscript.

Field-specific reporting

Please select the one below that is the best fit for your research. If you are not sure, read the appropriate sections before making your selection.

☒ Life sciences ☐ Behavioural & social sciences ☐ Ecological, evolutionary & environmental sciences

For a reference copy of the document with all sections, see nature.com/documents/nr-reporting-summary-flat.pdf

Life sciences study design

All studies must disclose on these points even when the disclosure is negative.

Sample size	49 whole genomes of endangered wild species. No more samples could be obtained.
Data exclusions	One sample (Nkuhene) was excluded due to very low quality, with 80% of read duplicates and 2X average coverage.
Replication	Not applicable, as the 49 genomes are independent individuals from the wild, as representatives of their respective populations.
Randomization	Not applicable, as these are wild individuals, and no experimental conditions apply.
Blinding	Not applicable, as no experimental conditions apply.

Reporting for specific materials, systems and methods

We require information from authors about some types of materials, experimental systems and methods used in many studies. Here, indicate whether each material, system or method listed is relevant to your study. If you are not sure if a list item applies to your research, read the appropriate section before selecting a response.

Materials & experimental systems

n/a	Involved in the study	
☒	☐	Antibodies
☒	☐	Eukaryotic cell lines
☒	☐	Palaeontology and archaeology
☒	☐	Animals and other organisms
☒	☐	Clinical data
☒	☐	Dual use research of concern

Methods

n/a	Involved in the study	
☒	☐	ChIP-seq
☒	☐	Flow cytometry
☒	☐	MRI-based neuroimaging
